# Direct comparison of high‐temporal‐resolution CINE MRI with Doppler ultrasound for assessment of diastolic dysfunction in mice

**DOI:** 10.1002/nbm.3763

**Published:** 2017-06-23

**Authors:** Thomas A. Roberts, Anthony N. Price, Laurence H. Jackson, Valerie Taylor, Anna L. David, Mark F. Lythgoe, Daniel J. Stuckey

**Affiliations:** ^1^ Centre for Advanced Biomedical Imaging University College London London UK; ^2^ Division of Imaging Sciences and Biomedical Engineering King's College London London UK; ^3^ Institute for Women's Health University College London London UK

**Keywords:** cardiovascular magnetic resonance, diastolic function, heart failure, high temporal resolution, preclinical imaging, ultrasound

## Abstract

Diastolic dysfunction is a sensitive early indicator of heart failure and can provide additional data to conventional measures of systolic function. Transmitral Doppler ultrasound, which measures the one‐dimensional flow of blood through the mitral valve, is currently the preferred method for the measurement of diastolic function, but the measurement of the left ventricular volume changes using high‐temporal‐resolution cinematic magnetic resonance imaging (CINE MRI) is an alternative approach which is emerging as a potentially more robust and user‐independent technique. Here, we investigated the performance of high‐temporal‐resolution CINE MRI and compared it with ultrasound for the detection of diastolic dysfunction in a mouse model of myocardial infarction. An in‐house, high‐temporal‐resolution, retrospectively gated CINE sequence was developed with a temporal resolution of 1 ms. Diastolic function in mice was assessed using a custom‐made, open‐source reconstruction package. Early (E) and late (A) left ventricular filling phases were easily identifiable, and these measurements were compared directly with high‐frequency, pulsed‐wave, Doppler ultrasound measurements of mitral valve inflow. A repeatability study established that high‐temporal‐resolution CINE MRI and Doppler ultrasound showed comparable accuracy when measuring E/A in normal control mice. However, when applied in a mouse model of myocardial infarction, high‐temporal‐resolution CINE MRI indicated diastolic heart failure (E/A = 0.94 ± 0.11), whereas ultrasound falsely detected normal cardiac function (E/A = 1.21 ± 0.11). The addition of high‐temporal‐resolution CINE MRI to preclinical imaging studies enhances the library of sequences available to cardiac researchers and potentially identifies diastolic heart failure early in disease progression.

**ABBREVIATIONS USED**
A: atrial/late‐filling phase
CINE: cinematic
CV: coefficient of variation
E: early‐filling phase
ECG: electrocardiography
EDV: end diastolic volume
EF: ejection fraction
ESV: end systolic volume
FA: flip angle
FLASH: fast low‐angle shot
FOV: field of view
Gd‐DTPA: gadolinium‐diethylenetriaminepentacetate
HFpEF: heart failure preserved ejection fraction
HTR: high temporal resolution
IR: inversion recovery
LAD: left anterior descending coronary artery
LGE: late gadolinium enhanced
LV: left ventricular/left ventricle
MRI: magnetic resonance imaging
NF: number of frames
NR: number of sequence repetitions
RC: repeatability coefficient
RF: radiofrequency
SEM: standard error of the mean
SV: stroke volume

## 1 INTRODUCTION

In the clinical setting, cardiac function is most commonly assessed using measures of contractile, systolic function, such as ejection fraction (EF), where a reduced EF (<50%) is a primary indicator of global systolic dysfunction.^1^, ^2^ However, heart failure with preserved EF (HFpEF) is now believed to be as common as heart failure with reduced EF.^3^ Between 40 and 71% of patients with HFpEF present with diastolic dysfunction,^4^ but although many treatments exist for tackling diseases associated with reduced EF, by comparison, the options available to patients with diastolic dysfunction are lacking. It has been shown that a multitude of pathophysiological processes either contribute to or lead to diastolic heart failure including – but not limited to – increased myocardial stiffness,^5^ impairment of calcium signalling during active diastolic relaxation,^6^ cardiac endothelial inflammation^7^ and mutations within mitochondria.^8^

Pulsed‐wave Doppler ultrasound imaging is the current standard for the diagnosis of diastolic dysfunction in patients,^1^, ^9^ but can be highly user dependent owing to the necessity for the manual placement of the ultrasound probe and acquisition voxel. MRI is emerging as a potentially superior alternative owing to its high spatial resolution and excellent tissue contrast,^10^, ^11^, ^12^ which could improve the detection of HFpEF in patients and help to unravel the myriad mechanisms underlying this multifarious disease through the use of animal models.

When using ultrasound, diastolic function is quantified by the measurement of blood velocity across the mitral valve, whereas, with MRI, quantification is based on the derivative of time–volume curves of the left ventricle (LV) blood pool chamber. With both modalities, the characteristic early‐filling (E) and atrial‐filling (A) phases can be identified. The ratio (E/A) of these two peaks is the metric most frequently used to diagnose diastolic dysfunction.^13^ In humans, an E/A ratio between 1.0 and 1.7 indicates normal diastolic function, whereas a ratio of less than 1.0 indicates impaired diastolic function related to impaired cardiac energetics. If E/A is greater 1.7, this can indicate restrictive filling associated with reduced myocardial compliance. For mice, these bounds are defined more loosely^14^: E/A greater than 1.0 equates to normal (or pseudo‐normal) LV filling, E/A less than 1.0 corresponds to abnormal LV relaxation and E much greater than A can indicate restricted filling.

In rodents, ultrasound remains the standard measure of diastolic dysfunction, but is also limited by user‐dependent transducer positioning, a small acoustic window, one‐dimensional acquisition and high heart rates, which can lead to merging of the E and A phases.^15^ Recently, two studies have demonstrated that cinematic magnetic resonance imaging (CINE MRI) can be used to measure cardiac diastolic function in mice through the use of high‐temporal‐resolution (HTR) techniques.^16^, ^17^ With upwards of 60–90 frames per cardiac cycle, HTR‐CINE acquisitions are able to resolve the filling peaks with a temporal resolution of almost 1 ms.

To date, however, HTR‐CINE techniques have not been compared with the ultrasound assessment of diastolic function. This comparison is important because the two modalities adopt intrinsically different approaches to the quantification of E/A: Doppler ultrasound measures transmitral blood flow directly, whereas HTR‐CINE methods measure local changes in LV volume.

Therefore, in this study, we directly compared a retrospectively gated HTR‐CINE sequence with ultrasound to validate the MRI approach, determine the repeatability of both techniques and examine how both methods perform when applied to a mouse model of heart failure. A single‐slice, retrospectively‐gated, HTR‐CINE sequence with an effective frame rate of 90 frames per cardiac cycle was used to measure diastolic function based on volumetric alterations in the LV, and compared with one‐dimensional, pulsed‐wave, Doppler ultrasound measures of mitral filling velocities. The variability and repeatability of both ultrasound and HTR‐CINE MRI were compared by repeat measurements of E/A ratios in normal control mice (*n* = 8). Following optimisation of the MRI protocol, both modalities were then applied to the measurement of diastolic function in mice after surgically induced myocardial infarction (*n* = 13).

## 2 METHODS

### 2.1 Ethics statement

All animal studies were approved by the University College London Biological Services Ethical Review Committee and licensed under the UK Home Office regulations and the Guidance for the Operation of Animals (Scientific Procedures) Act 1986 (Home Office, London, UK).

### 2.2 Mouse model of myocardial infarction

Mice were anaesthetised in 4% isoflurane, intubated and maintained at 1.5% isoflurane in oxygen. Analgesia (0.1 mg/kg intramuscular buprenorphine) was administered before and 6 and 24 h after surgery. Body temperature was monitored using a rectal thermometer and maintained using a heated operating table. Open‐chest surgery was performed to gain access to the heart. The left anterior descending coronary artery (LAD) was permanently occluded using a 5–0 prolene suture approximately 2 mm below the origin. All surgical openings were closed and the animal was recovered.

### 2.3 MRI setup

MRI measurements were performed on a 9.4‐T horizontal bore scanner (Agilent Technologies, Santa Clara, CA, USA) with a 115/60/HD 1000‐mT/m gradient insert and a 35‐mm‐volume radiofrequency (RF) coil (RAPID biomed, Ripmar, Germany). Mice were anaesthetised with 1.5–2.0% isoflurane in 2 L/min oxygen and positioned supine in a cradle for imaging. Body temperature was regulated using a hot water system and monitored using a rectal probe. Respiration was monitored using a neonatal apnoea pad taped to the abdomen of the animal. Electrocardiography (ECG) was monitored using a subcutaneous three‐lead electrode configuration (Model 1030 Monitoring & Gating System, SA Instruments, Stony Brook, NY, USA), which was robust to interference from the imaging gradients. Two of the electrodes were placed at the base of each fore leg and the final electrode was placed in the right flank of the mouse. For gadolinium‐based experiments, an intraperitoneal infusion line was inserted into the mouse.

### 2.4 Ultrasound setup

Ultrasound measurements were performed on a Vevo 2100 system (VisualSonics, California, CA, USA) using an MS550D 30‐MHz transducer (VisualSonics). Mice were anaesthetised with 1.5–2.0% isoflurane in 2 L/min oxygen and positioned supine on a physiological monitoring platform which simultaneously regulated body temperature, whilst measuring respiration and ECG traces. Prior to imaging, hair was removed from the chest to reduce the attenuation of the ultrasound signal.

### 2.5 Study design

Two cohorts of 10–12‐week‐old male C57BL/6 mice were imaged using both ultrasound and HTR‐CINE MRI. In group one, control mice were imaged to study the repeatability of both modalities. First, mice were imaged with ultrasound, then taken for MRI with a standard prospective CINE sequence and an HTR‐CINE sequence. Animals were then removed and repositioned in the scanner before both MRI sequences were repeated. This was followed by a second trial of ultrasound. In total, *n* = 6 mice were imaged with both modalities; two mice died under anaesthesia before the second trial of ultrasound. All eight mice were imaged with repeat measures of MRI. Each mouse in group one was imaged for a maximum total of 2.5 h.

In group two, *n* = 13 mice underwent surgically induced permanent occlusion myocardial infarction (as described above). Four weeks later, all animals were imaged with ultrasound, followed by standard CINE and HTR‐CINE imaging. Following these protocols, infarcted animals were injected with gadolinium contrast agent and imaged with an inversion recovery late‐gadolinium‐enhanced (IR LGE) sequence for infarct size quantification.^18^ Each mouse in group two was imaged for a maximum total of 1 h.

### 2.6 Determination of optimum slice location for single‐slice MRI

The acquisition of a full stack of HTR‐CINE images through the heart was not feasible because of experimental time constraints. Therefore, in the control cohort of mice, the HTR‐CINE sequence was applied at three different short‐axis slice locations and compared with ultrasound measurements of transmitral valve blood flow. The slice locations were defined as follows: basal (the first slice below the mitral valve), mid‐ventricular and apical (the next two contiguous slices, respectively).

### 2.7 Prospective CINE imaging for the measurement of systolic function

Parameters of systolic cardiac function were measured using a spoiled, prospectively ECG‐gated, fast low‐angle shot (FLASH) sequence as described in Stuckey et al.^19^ CINE images consisting of 18–24 frames per cardiac cycle – depending on the heart rate of the animal (Table 1).– were acquired in a short‐axis orientation with the following parameters: TR = 5 ms; TE = 1.21 ms; flip angle (FA), 15°; data matrix, 128^2^; field of view (FOV), 25.6 mm^2^; slice thickness, 1 mm; number of slices, 9–12 (depending on the animal). Manual segmentation of the LV blood pool in all slices was used to calculate the end systolic volume (ESV), end diastolic volume (EDV), stroke volume (SV) and EF.

**Table 1:** Physiological parameters (mean ± standard deviation) for both trials in the control group of mice and for the infarcted group of mice. Body temperature was maintained at physiological temperature

	Control – Trial 1	Control – Trial 2	Infarct
Heart rate (beats/min)	588 ± 38	563 ± 39	590 ± 38
Respiration rate (breaths/min)	58 ± 12	49 ± 6	51 ± 11

### 2.8 HTR retrospective CINE imaging for the measurement of diastolic function

The same single‐slice FLASH sequence as described above was used for the measurement of diastolic function, except that it was amended to run without respiration or ECG gating and with a lower repetition time, TR = 3.15 ms. Instead of prospective gating, the sequence was run repeatedly for a total (NR) of 1400 times, equivalent to 9.5 min. During this time, all respiration, ECG and RF events were recorded using a Power1401 datalogger in combination with Spike2 software (Cambridge Electronic Design, Cambridge, UK) for retrospective reconstruction. HTR‐CINE images were then created offline in MATLAB (Mathworks, Natick, MA, USA) using custom‐made scripts which reconstructed *k*‐space based on the timings of the events recorded by the datalogger (Figure S1a). Simulations (not shown) determined that NR = 1400 was sufficient to generate HTR‐CINEs with 90 frames (NF) per cardiac cycle, with at least 99% of *k*‐space filled with a minimum of three averages (Figure S1b). Further details of the custom‐made, retrospective, HTR‐CINE sequence and how to download the reconstruction code are given in Supporting Information.

Time–volume curves were generated by manual segmentation of the LV blood pool. The blood pool volume within a slice was calculated based on the product of the in‐plane voxel area, slice thickness and number of pixels in the segmented region. The first derivative of the time–volume curve was taken to calculate cardiac filling rates. These curves were used to determine the E and A peaks by manual measurement. For further analysis, the volume curves from each of the three slices were averaged together to create a combined curve which represented the whole imaging volume. E and A were also determined from this combined curve and compared with ultrasound.

### 2.9 LGE MRI for the imaging of myocardial infarcts

Myocardial infarct size was measured using a multi‐slice gradient echo IR LGE sequence, described previously.^18^ In brief, 0.6 mmol/kg gadolinium‐diethylenetriaminepentacetate (Gd‐DTPA) (Magnevist, Schering AG, Berlin, Germany) was injected via an intraperitoneal infusion line 10 min prior to imaging. Imaging parameters were as follows: TR = 3.1 ms; TE = 1.21 ms; FA, 90°; TI = ~4 × R–R interval; data matrix, 128^2^; FOV, 25.6 mm^2^; slice thickness, 1 mm.

### 2.10 Ultrasound imaging for the measurement of diastolic function

For the assessment of diastolic function, colour B‐mode‐guided, pulsed‐wave, Doppler mode ultrasound was used to measure mitral inflow in an apical four‐chamber view of the heart. E and A peaks were measured from three consecutive heart beats, and the mean E/A ratio was calculated for each animal.

### 2.11 Statistics

The coefficient of variation (CV) is a normalised measure of data variability given by the ratio of the standard deviation *σ* to the mean *μ*:

$$\mathrm{CV}=\frac{\sigma }{\mu }\times 100\%$$

CV was used to calculate the variability in the measurements of E/A between repeat trials in the control cohort using both ultrasound and HTR‐CINE MRI. For further comparison, CV was also calculated for measures of systolic function: EF, ESV, EDV and SV.

Bland–Altman plots were produced to examine the repeatability of E/A estimates using both modalities. The Bland–Altman repeatability coefficient (RC) was calculated for each plot, which represents the 95% confidence interval of the difference in the two trials. RC is given by:

$$\mathrm{RC}=1.96\sqrt{\frac{\sum \left(\Delta F^{2}\right)}{n-1}}\times \frac{100\%}{\bar{F}}$$

where Δ*F* is the change in estimates of E/A between trials and $\bar{F}$ is the mean estimate of E/A between trials. Similarly, RC was calculated for measures of EF and SV. For interpretation, RC provides an indication of the change required to observe a difference above variation.

Bland–Altman plots were also used to visualise inter‐modality variation. Spearman's correlation coefficient was calculated to examine the level of agreement between inter‐modality measurements of E/A in both the naïve and infarcted cohorts of mice.

## 3 RESULTS

### 3.1 Measurements of standard systolic function parameters

**Figure 1:**
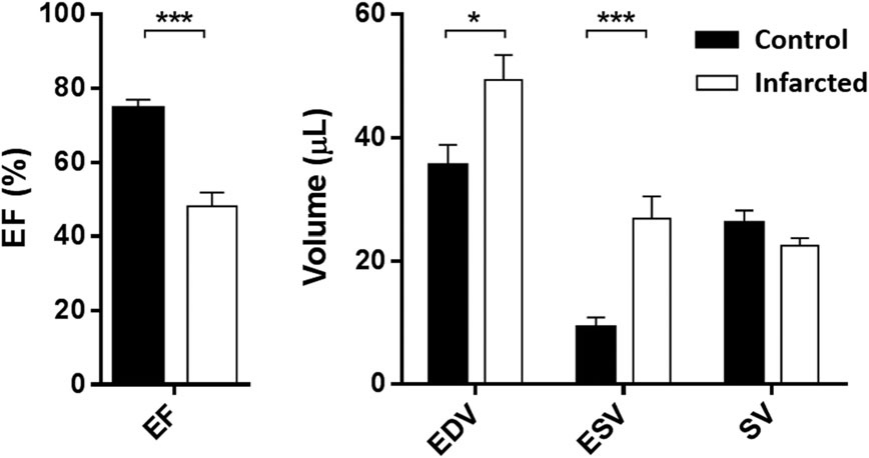
Comparison of standard cinematic (CINE)‐derived cardiac systolic parameters in the control (*n* = 8) and infarcted (*n* = 13) groups. Error bars indicate standard error of the mean. Mann–Whitney *U*‐tests performed between groups for each parameter: ***p < 0.001; *p < 0.05. EDV, end diastolic volume; EF, ejection fraction; ESV, end systolic volume; SV, stroke volume

Eight control mice and 13 infarcted mice were imaged with standard CINE imaging (Videos S1 and S2) to determine the systolic parameters of cardiac function. Figure 1 shows the mean values [and standard error of the mean (SEM)] of four standard systolic parameters. As expected, EF was 36% lower in the infarcted cohort (*p* = 0.0002) compared with the control animals. EDV was 39% higher (*p* = 0.019), ESV was more than 200% higher (*p* = 0.0006) and SV was not significantly different between the cohorts (*p* = 0.19).

### 3.2 *In vivo* measurement of diastolic function

**Figure 2:**
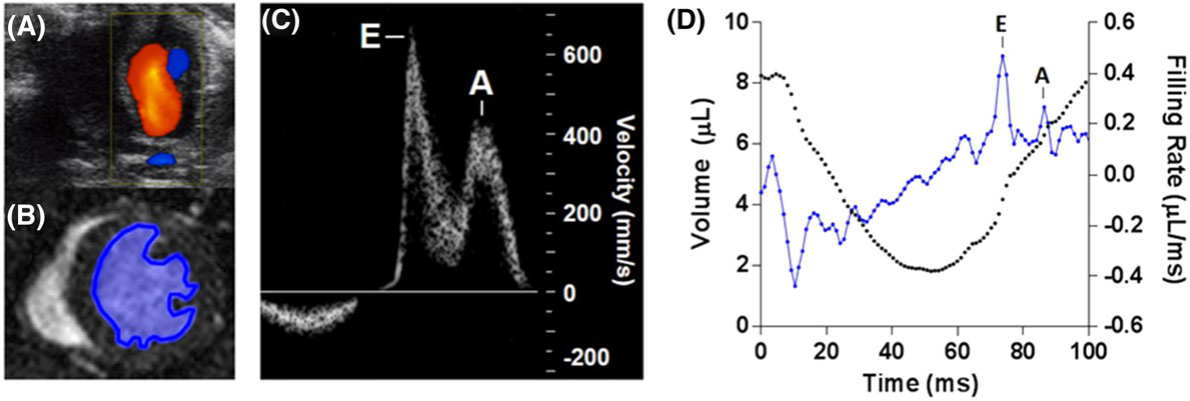
Representative filling rate curves from the same mouse measured using ultrasound and high‐temporal‐resolution cinematic magnetic resonance imaging (HTR‐CINE MRI) showing early‐filling (E) and atrial/late‐filling (A) peaks. (A) ultrasound image of mitral blood flow showing Doppler measurements overlaid. (B) HTR‐CINE image showing left ventricle blood pool segmentation. (C) ultrasound filling curve of blood velocity through the mitral valve. (D) HTR‐CINE‐derived left ventricle volume curve (black) and filling rate curve (blue)

Figure 2 shows representative ultrasound and MR images acquired in the same control mouse. For the assessment of diastolic function using ultrasound, pulsed‐wave measurements were made at the site of peak inflow identified in the colour B‐mode apical four‐chamber view. The E and A peaks were measured and averaged across three consecutive filling curves (Figure 2c). For HTR‐CINE MRI analysis (Video S3), the LV blood pool was manually segmented in every frame of the cardiac cycle to generate a time–volume curve, as shown in Figure 2d (black). The filling rate curve (blue) was then calculated by differentiation of the time–volume curve. Distinct E and A peaks could be resolved using both modalities.

### 3.3 Variability and repeatability of parameter measurements

**Figure 3:**
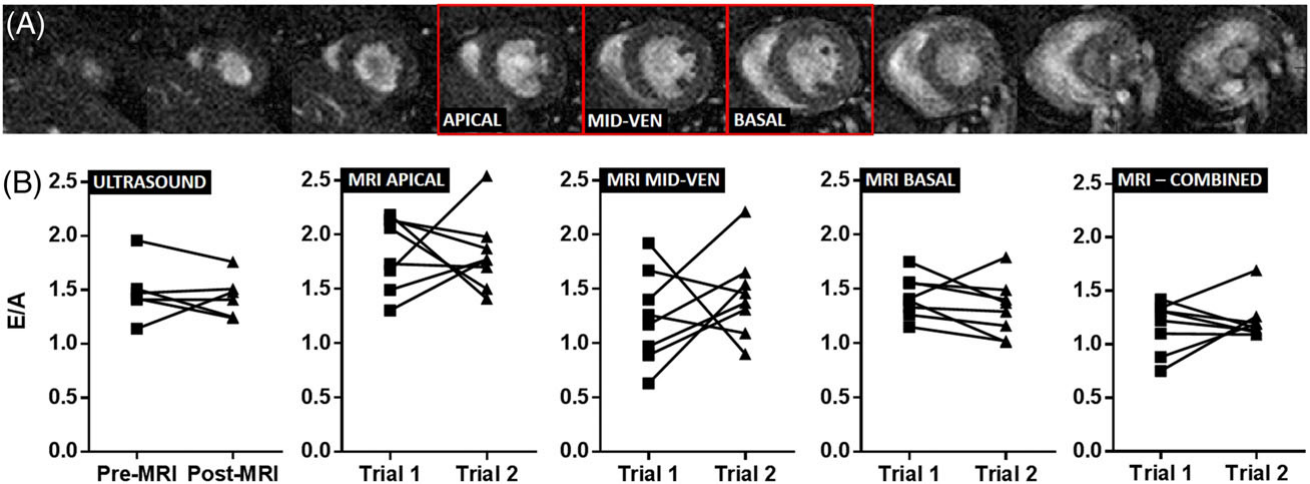
(A) standard prospective cinematic (CINE) stack showing short‐axis images through a control mouse heart at diastole. Red boxes denote slices chosen for the determination of the most optimum slice in early‐filling to atrial/late‐filling ratio (E/A) measurements. (B) raw data from repeated measures of E/A in control mice using ultrasound and magnetic resonance imaging (MRI) at three different slice locations (apical, mid‐ventricle and basal) and when all three slices were combined (for all repeat measurements, *p* > 0.05, Wilcoxon signed‐rank test)

Figure 3a shows a standard prospective CINE stack through a mouse heart – the slices outlined in red represent the three different short‐axis slice locations (defined as basal, mid‐ventricular and apical) examined for optimisation of the single‐slice HTR‐CINE protocol. Figure 3b shows raw E/A measurements assessed in repeated trials using both modalities. The mean E/A ratio (±SEM) measured using ultrasound was 1.41 ± 0.06. The mean E/A ratios measured using HTR‐CINE MRI were 1.42 ± 0.10 in mid‐ventricle slices, 1.39 ± 0.06 in basal slices and 1.23 ± 0.05 when all slices were combined and analysed as a single time–volume curve. These measurements were all in the range of normal heart function based on ultrasound metrics of E/A in mice (>1.0) and humans (1.0–1.7). In apical slices, this value increased to 1.84 ± 0.08. There were no significant differences between repeat trials using either modality, regardless of slice location when using MRI (*p* > 0.05, Wilcoxon signed‐rank test).

Visual inspection of the raw data (Figure 3b) indicated that there was noticeable variability in HTR‐CINE MRI measurements depending on the choice of slice location. For example, there was a larger spread in E/A values measured in mid‐ventricle slices relative to basal slices. Correspondingly, measurements of the CV (Table 2) showed that ultrasound had the lowest variability (CV = 8.5 ± 2.8%), followed by HTR‐CINE MRI measurements in a basal slice location (CV = 10.4 ± 2.6%). Variability increased by approximately a factor of two using an apical slice location and a factor of three using a mid‐ventricle slice location, compared with ultrasound. When all slices were combined, the variability (CV = 14.0 ± 3.9%) was higher than ultrasound and basal slice HTR‐CINE measurements.

**Table 2:** Mean parameter values for both trials in the control group and coefficient of variation [CV (± SEM)]. Systolic cardiac parameters were measured with a standard CINE imaging protocol. E/A measurements were made using ultrasound and HTR‐CINE imaging at different slice locations in the heart

Systolic parameter	Mean – Trial 1	Mean – Trial 2	CV (%)
Ejection fraction (EF) %	74.7 ± 2.2	75.2 ± 1.9	3.4 ± 0.8
End diastolic volume (EDV) μL	35.6 ± 3.3	38.1 ± 3.3	8.6 ± 2.3
End systolic volume (ESV) μL	9.3 ± 1.5	9.6 ± 1.2	16.0 ± 4.1
Stroke volume (SV) μL	26.2 ± 2.0	28.5 ± 2.2	7.4 ± 1.9
**Method of E/A measurement**			
HTR‐CINE MRI – Apical	1.84 ± 0.12	1.82 ± 0.12	16.4 ± 3.9
HTR‐CINE MRI – Mid‐ventricle	1.24 ± 0.15	1.44 ± 0.14	29.6 ± 6.3
HTR‐CINE MRI – Basal	1.43 ± 0.07	1.32 ± 0.09	10.4 ± 2.6
HTR‐CINE MRI – Combined	1.17 ± 0.08	1.23 ± 0.07	14.0 ± 3.9
Ultrasound	1.49 ± 0.11	1.36 ± 0.08	8.5 ± 2.8

A, atrial/late‐filling phase; CINE, cinematic; CV, coefficient of variation; E, early‐filling phase; HTR, high temporal resolution; SEM, standard error of the mean.

For comparison, Table 2 also shows CV values for various systolic cardiac parameters alongside CV values of E/A measurements. As expected, EF was the parameter with the lowest variability (CV = 3.4 ± 0.8%) because it is calculated from a combination of the other parameters. ESV showed the highest variability (CV = 16.0 ± 4.1%) as accurate blood pool segmentation is difficult when the LV chamber volume is at its smallest. Variability in E/A measurements using ultrasound and HTR‐CINE MRI in a basal slice were closest to measurements of EDV (CV = 8.6 ± 2.3%).

**Figure 4:**
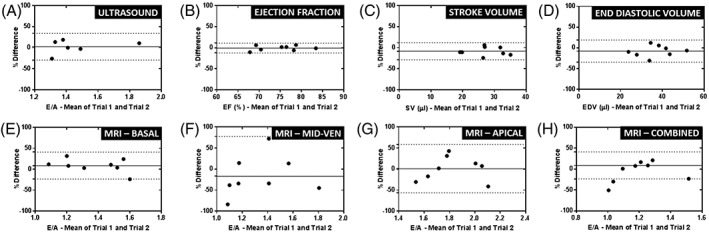
Bland–Altman plots showing repeatability of the early‐filling to atrial/late‐filling ratio (E/A) [and more routinely assessed cardiac parameters – Ejection fraction (EF), stroke volume (SV), end diastolic volume (EDV)] in the naïve cohort. (A) E/A measured using ultrasound. (B–D) EF, SV and EDV measured using magnetic resonance imaging (MRI). (E–H) E/A measured using high‐temporal‐resolution cinematic MRI (HTR‐CINE MRI) at different locations in the heart (basal, mid‐ventricle and apical) and with all three slices combined. Full lines represent the mean percentage difference between trials and broken lines represent 95% confidence intervals (±1.96 × standard deviation of the difference between the trials)

Bland–Altman analysis was also carried out to examine the repeatability of E/A measurements (Figure 4). RC for ultrasound was 23%. This increased slightly to 25% for MRI measurements in a basal slice location. RC was much higher using mid‐ventricle (37%) and apical (94%) slice locations. With all slices combined, RC was 47%. For systolic cardiac parameters, RC was lowest at 9% for measurements of EF. This increased to 21% for SV and 24% for EDV measurements.

Inter‐modality analysis demonstrated that the best agreement was between ultrasound measurements of E/A and basal slices using HTR‐CINE MRI (Figure S2a). Spearman's coefficient was only significantly correlated (*ρ*_s_ = 0.54, *p* = 0.04) when comparing ultrasound with basal slices. Furthermore, the bias was small at 2% and the SD of the bias at 15% was the lowest of all the slice positions.

The variability, repeatability and inter‐modality analysis of E/A measurements indicated that, for subsequent studies, basal slice locations should be used to measure E/A with HTR‐CINE MRI.

### 3.4 Assessment of the infarct size and diastolic function in infarcted mouse hearts

**Figure 5:**
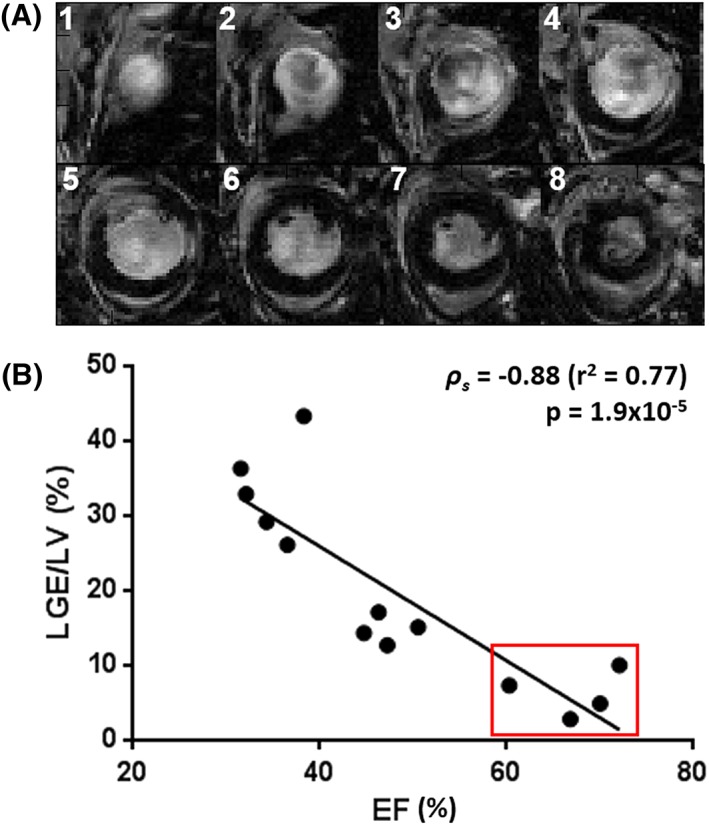
(A) short‐axis view of late‐gadolinium‐enhanced magnetic resonance imaging (LGE MRI) in an infarcted mouse heart (1, most apical slice; 8, most basal slice). Hyperintensity within the myocardium corresponds to infarcted tissue. (B) correlation of ejection fraction (EF) and area of gadolinium enhancement in the infarcted cohort for the determination of infarct severity. The red box denotes animals with minor cardiac impairment [EF > 50%, total infarct volume as a percentage of the left ventricular volume (LGE/LV) < 10%] where surgery was ineffective. These animals were excluded from subsequent analysis. LV, left ventricle

Four weeks after myocardial infarction, 13 mice were imaged using standard CINE imaging, HTR‐CINE imaging (Video S4) and LGE IR MRI to determine infarct size. Figure 5a shows a representative stack of slices acquired in an infarcted mouse heart. Normal myocardial tissue is dark. Segmentation of the hyperintense infarcted tissue enabled the calculation of the total infarct volume as a percentage of the LV volume (LGE/LV, %). The mean total infarct volume across all 13 mice was 19.4 ± 3.6% (SEM). As expected, an inversely proportional relationship was observed between EF and infarct size (Figure 5b), which showed a significant correlation (Spearman's correlation coefficient, *ρ*_s_ = −0.88, *p* = 0.000019).

However, a broad range of infarct volumes was measured, from 3% to 43%, indicating that surgery was not always successful. Four mice from the cohort were identified as having only mild cardiac impairment, defined as EF > 50% and LGE/LV < 10%, where there was either normal or slightly reduced EF compared with the control cohort, in combination with only minor gadolinium contrast enhancement within the myocardium. Consequently, these mice in the cohort were excluded from subsequent analysis (red outline in Figure 5b). The significant correlation between EF and infarct size was retained when removing these four mice from the analysis (Spearman's correlation coefficient, *ρ*_s_ = −0.75, *p* = 0.02).

**Figure 6:**
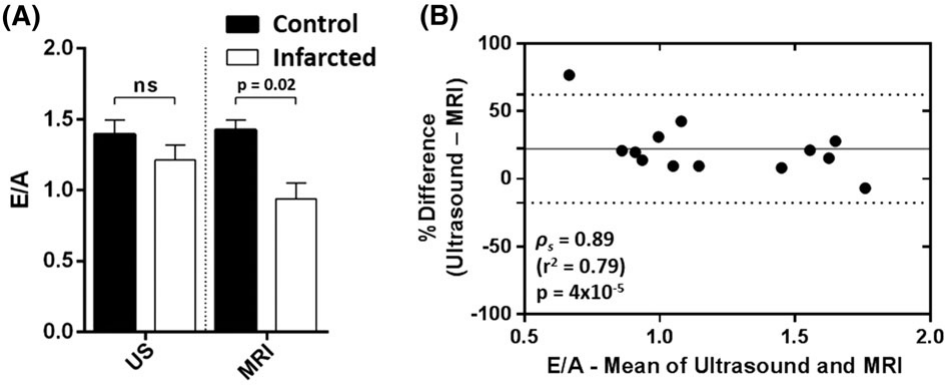
(A) comparison of early‐filling to atrial/late‐filling ratio (E/A) measurements determined by ultrasound (US) and high‐temporal‐resolution cinematic magnetic resonance imaging (HTR‐CINE MRI) in the infarcted cohort of mice with ejection fraction (EF) < 50% and total infarct volume as a percentage of the left ventricular volume (LGE/LV) > 10% (*n* = 9). There was a significant difference in E/A between control and infarcted mice when measured with MRI (*p* = 0.02, Mann–Whitney *U*‐test), which was not observed with ultrasound (*p* = 0.15). Ns, not significant. (B) Bland–Altman plot of E/A between ultrasound and MRI across all 13 animals in the infarcted cohort. E/A values tended to be lower when assessed with MRI relative to ultrasound (bias, 22%). Spearman's coefficient showed that the two modalities were strongly correlated (*ρ*_s_ = 0.89, *p* = 0.00004)

E/A ratios were assessed in the infarcted cohort (*n* = 9) using ultrasound and basal slice measurements with HTR‐CINE MRI. Figure 6a shows the mean E/A (and SEM) values in the infarcted cohort compared with the control group (data taken from post‐MRI ultrasound scans and trial 2 basal MRI scans only). With ultrasound, a reduction in E/A of 11% was observed from 1.36 ± 0.08 in the control group to 1.21 ± 0.11 in the infarcted group. This was lower than the RC for ultrasound (23%) and the difference between the groups was not significant (*p* = 0.15, Mann–Whitney *U*‐test). With MRI, a reduction in mean E/A of 29% was observed from 1.32 ± 0.09 in the control group to 0.94 ± 0.11 in the infarcted group. This was above the RC value for MRI (25%, basal slices) and the difference between groups was significant (*p* = 0.02, Mann–Whitney *U*‐test).

Bland–Altman analysis of the E/A ratios between the two modalities across the full infarcted cohort (Figure 6b, *n* = 13, including mice with minor cardiac impairment) showed a positive bias (22%): there was a tendency for MRI measures of E/A to be lower than ultrasound measurements. Calculation of Spearman's coefficient (*ρ*_s_ = 0.89, *p* = 0.00004) showed that the two modalities were strongly correlated.

## 4 DISCUSSION

HTR MRI techniques are emerging as a useful alternative to pulsed‐wave Doppler ultrasound for the examination of diastolic cardiac function in the mouse. Yet, despite the intrinsically different approaches to the quantification of E/A, this is the first study to investigate the value of these methods by direct comparison.

The E/A ratio was used as the metric for the quantification of diastolic function as it has been proven to be a sensitive biomarker of myocardial energetics and compliance. However, ultrasound and MRI derive this parameter in different ways: pulsed‐wave Doppler ultrasound measures a one‐dimensional profile of blood inflow across the mitral valve, whereas HTR‐CINE MRI measures the dilation of the LV chamber in a single slice. Time considerations and variation in relaxation time across the ventricular long axis mean that full chamber coverage is inappropriate for the HTR‐CINE sequence. Therefore, it was important to choose and establish the slice position with the lowest variability in measurements of the E/A ratio. The mean E/A ratio when using apical slices was high in the control cohort (1.84 ± 0.08), potentially giving a false indication of cardiac impairment based on a human metric of diastolic function.^13^ Although a mid‐ventricle slice location produced a mean E/A ratio indicative of normal function (1.42 ± 0.10), the variability (CV = 16.4%) was much greater than when using a basal slice location (CV = 10.4%). This range of variability illustrated the importance of slice location when using a single‐slice acquisition. Basally located slices were most comparable with ultrasound measurements, most probably because this region is closest to the mitral valve where filling originates, resulting in the sharpest filling peaks. In slices located more distally from the mitral valve, the velocity of the inflowing blood is decreased and delayed, causing a smoothing of the filling peaks, making them harder to define.

The repeatability of E/A measurements was comparable between modalities in the control cohort of mice. Ultrasound had a slightly lower RC – by 2% – compared with basal HTR‐CINE MRI. For comparison with routinely measured systolic parameters, RC of EF and SV were calculated. EF was much lower (RC = 9%) than E/A, as expected, but SV (RC = 21%) was similar to measures of E/A, illustrating that measurements of diastolic function using both modalities are comparably consistent with well‐established, accepted measures of systolic function.

When the three slices were combined to produce a single time–volume curve, CV and RC were higher than both ultrasound and single‐slice HTR‐CINE in a basal location, most probably because the increased variability of the mid‐ventricular and apical slices (compared with basal) is averaged into the measurement.

The low variability, strong repeatability and good inter‐modality agreement of HTR‐CINE MRI in a basal location were the determining factors for the use of this slice location when imaging the infarcted cohort. In addition, it made sense to assess diastolic function in this location because changes in the LV filling curves will be caused by impaired, but functional, myocardium, rather than directly related to the extent of akinetic scar tissue within the chosen slice, as would be the case in more apical locations. For these reasons, we recommend that future studies wanting to employ MRI to measure diastolic function in mouse hearts use a slice located as close to the mitral valve as possible, whilst avoiding partial volume effects with the left atrium.

The mean E/A ratios measured in the control cohort using a basal slice location for HTR‐CINE MRI were comparable with those determined by other authors. For example, experiments by Coolen et al.^17^ in non‐diabetic control mice measured mean E/A = 1.51 ± 0.08 compared with our value of 1.39 ± 0.06. Similarly, Daniels et al.^20^ measured E/A = 1.5 ± 0.1 using ultrasound, also in non‐diabetic mice. An earlier study by Stuckey et al.^16^ found E/A to be lower at 1.06 ± 0.06; however, this value is still within the range of normal cardiac function.

In the second study, the two modalities were applied to a cohort of mice with myocardial infarction to examine the performance of both techniques for the detection of diastolic dysfunction. We chose to assess diastolic function in myocardial infarction because we have expertise in this mouse model and it is the most frequently used model of heart disease. The use of this mouse model is a limitation of the current study as ideally it would be most valuable and interesting to examine diastolic dysfunction in other subtler disease models, such as diabetic mouse models in which systolic function is better preserved.

The infarcted cohort was imaged with standard CINE imaging as well as IR LGE imaging to determine EF and infarct size. Analysis revealed that the cohort exhibited a range of infarct severities, with some having minimal cardiac impairment; hence, four mice were excluded from the analysis of diastolic dysfunction. On average, infarcted mice exhibited reduced E/A ratios compared with healthy mice. With ultrasound, there was an 11% decrease in mean E/A from 1.36 ± 0.08 in the control group to 1.21 ± 0.11 in the infarcted group. Correspondingly, this still indicates normal cardiac function (E/A > 1.0). With MRI, the mean E/A in the infarcted group was 29% lower at 0.94 ± 0.11, on average suggesting cardiac impairment (E/A < 1.0). These results indicate that HTR‐CINE was able to detect abnormal diastolic function, whereas ultrasound was not able to detect this difference. The reason for this difference may possibly be attributed to the quantification of E/A in different ways by the two modalities; the morphological change measured by MRI may be a more direct measure of myocardial relaxation than ultrasound measurement of blood velocity. These morphological changes may be more pronounced than alterations to flow when impairment is low, such as in the relatively small infarcts used in this study.

Alternative MRI methods, including phase contrast velocity encoding of flow across the mitral valve^21^, ^22^ and tissue tagging for strain analysis of myocardial motion,^23^, ^24^ show great promise for the monitoring of diastolic function; however, the data acquisition protocols are relatively complex compared with the modifications required to convert a routine CINE sequence into an HTR acquisition, which essentially involves repeating a standard sequence many times. Prospective CINE imaging for the measurement of diastolic function requires less post‐processing than retrospective acquisitions, but frame rates are typically capped at <60 frames because of hardware constraints. Consequently, this reduction in temporal resolution means that there is less sampling of the E and A peaks. Prospective strategies also require triggering every second R‐wave to ensure that the full cardiac cycle is imaged – this results in data redundancy as the frames acquired in the second R–R interval are typically discarded. Retrospective reconstruction is also less susceptible to gating errors, such as mistimed or missed R‐waves, which can be adjusted or reinstated during HTR‐CINE data processing.

In summary, this study has shown, for the first time, that HTR‐CINE MRI can measure diastolic function in normal mice with an accuracy and repeatability comparable with the accepted standard of pulsed‐wave Doppler ultrasound. When applied to a mouse model of myocardial infarction, HTR‐CINE MRI detected mild heart failure, whereas measurement with ultrasound did not reach significance.

## Supporting information


**Figure S1** Supporting info item

**Figure S2** Supporting info item

**Supplementary Video S1:** Example standard CINE movie from a basal slice location in a normal mouse. (Supplementary_Video_S1_Normal_20frames.avi)

**Supplementary Video S2:** Example standard CINE movie from a basal slice location in an infarcted mouse. (Supplementary_Video_S2_Infarct_20frames.avi)

**Supplementary Video S3:** Example retrospective HTR‐CINE movie (showing 90 frames through the cardiac cycle) from the same animal and slice location as in Supporting Video S1. (Supplementary_Video_S3_Normal_90frames.avi)

**Supplementary Video S4:** Example retrospective HTR‐CINE movie (showing 90 frames through the cardiac cycle) from the same animal and slice location as in Supporting Video S2. (Supplementary_Video_S4_Infarct_90frames.avi)
