# Supplementary figures and images for: Direct comparison of high‐temporal‐resolution CINE MRI with Doppler ultrasound for assessment of diastolic dysfunction in mice
Source: NMR Biomed. 2017 Jun 23;30(10):e3763. doi: 10.1002/nbm.3763 (PMC5638074; doi:10.1002/nbm.3763)

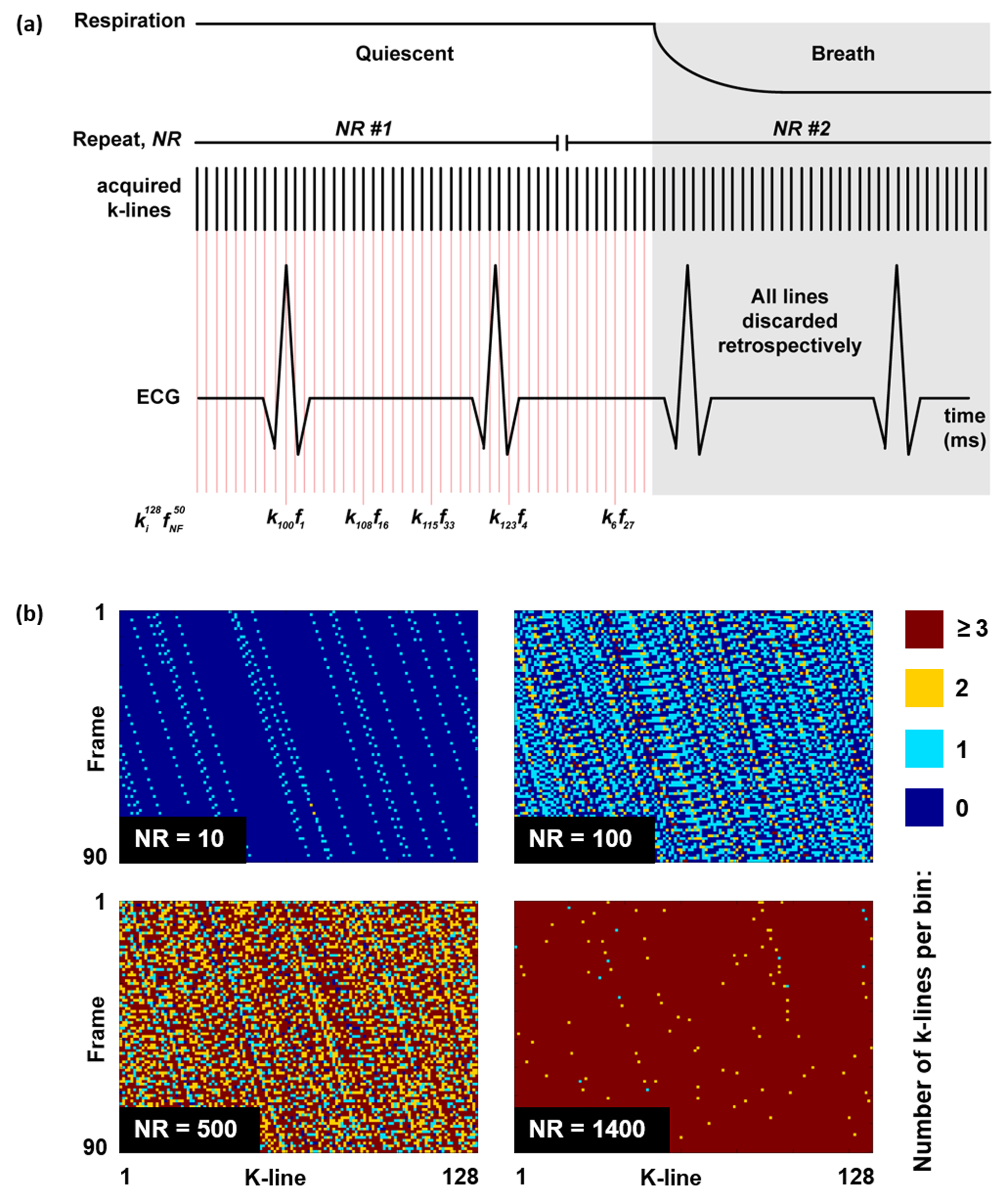

Supplement: Supplementary file 1 — Figure S1 Supporting info item [file NBM-30-na-s001.tif]

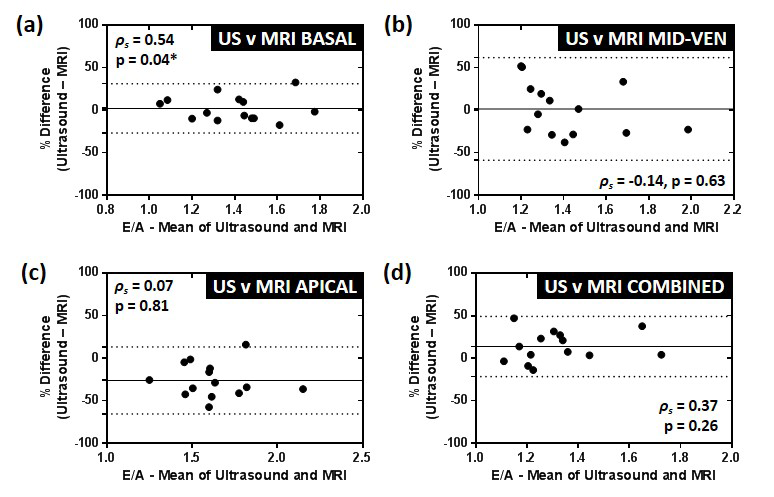

Supplement: Supplementary file 2 — Figure S2 Supporting info item [file NBM-30-na-s002.tif]
